# Reply to Sun: Dynamic and accessible pharmacogenomic results: a response to pharmacogenomic testing for antidepressant treatment selection

**DOI:** 10.1038/s41386-024-01815-4

**Published:** 2024-02-15

**Authors:** Mark A. Frye, Charles B. Nemeroff

**Affiliations:** 1 Department of Psychiatry & Psychology, Mayo Clinic, Mayo Clinic Alix School of Medicine, Rochester, MN USA; 2 Department of Psychiatry & Behavioral Sciences, University of Texas at Austin Dell Medical School, Austin, TX USA

**Keywords:** Neuroscience, Psychology

We thank Dr. Sun and colleagues for their timely, clinically relevant perspective on how dynamic access to pharmacogenomic testing results is critical to ensure this new knowledge can optimize the potential to inform clinical decision making. There is agreement that for clinical decision support tools to have maximal utility, electronic health record (EHR) functionality must focus on an electronic interface, ideally not an electronic file, which facilitates easy access to the data beyond the ordering clinician and allows for updates of new pharmacogenomic variants and antidepressants. Hard copy paper reports loaded into a media section or static laboratory reports without capacity to add new knowledge will quickly become non-current and likely lost in the EHR.

Recent survey research has investigated primary care providers’ knowledge, clinical use, and impression of clinician decision support tools, both pharmacogenomic testing results and automatic alerts of genetic variants viewed as clinically actionable. Overall, more than 50% of clinicians did not expect to use pharmacogenomic information in their future practice and similarly, 50% found alerts (i.e., 2D6 poor metabolizer phenotype) confusing and difficult to find additional information [1]. Concerted efforts towards a more efficient user-friendly interface are highly encouraged as single genetic variants that are categorized as poor or ultra-rapid metabolizer phenotypes are clinically actionable today; both cytochrome P450 2D6 poor and ultra-rapid metabolizer phenotypes have been associated with significant higher rates of either poor pain control or adverse drug related event to opioid medication [2] and risk of hospitalization and emergency department (ED) visit (ultra-rapid only) [3]. While these studies are not specifically psychiatric patients or antidepressants, future mechanistic studies of the association of these variants and hospital outcome measures usually monitored by the Centers for Medicare & Medicaid Services (CMS) are valuable at both and the individual and population-based level.

While there are certainly single pharmacodynamic genetic variants for black box warnings that are relevant to clinical practice in mood disorders today (i.e. Stephens Johnsons Syndrome, HLA-B*1502 variation, carbamazepine and QTc prolongation, 2D6 poor metabolizer phenotype, fluoxetine), clinical studies of decision support tools for a full cadre of various antidepressants do not reach the level of clinical evidence. Sun and colleagues reference a 2019 meta-analysis of pharmacogenomic guided antidepressant selection vs treatment as usual including five randomized controlled trials that encompassed 1737 participants [4]. The results of this meta-analysis suggested the benefit of pharmacogenomic testing on remission rates [relative risk 1.71 (95% CI: 1.17–2.48; *p* = 0.005)]. Since this publication, however, PRIME CARE, the largest controlled study to date that enrolled 1944 participants, more than the five studies of the meta-analysis combined, did not achieve significance on their co-primary outcome measure -predicted drug-gene interaction prescription and remission of depression [5]. Moreover, a comprehensive review of pharmacogenomic testing to predict antidepressant efficacy or side effects concluded that there is no current utility for any of the commercially available tests [6].

New drug therapies and ever evolving genetic variants and technology will dynamically advance current conventional decision support tools. When and how to scale these tools in an optimal EHR interface for treatment selection of antidepressants for major depressive disorder remain unclear.
